# Delayed Healthcare Due to Cost Among Adults with Multimorbidity in the United States

**DOI:** 10.3390/healthcare12222271

**Published:** 2024-11-14

**Authors:** Chidimma Doris Azubuike, Oluwatobi Abel Alawode

**Affiliations:** 1 Department of Pharmaceutical Outcomes and Policy, University of Florida, Gainesville, FL 32603, USA; azubuikechidimma@ufl.edu; 2 Department of Sociology and Criminology & Law, University of Florida, Gainesville, FL 32611, USA

**Keywords:** chronic disease, delayed healthcare, healthcare access, healthcare utilization, multimorbidity

## Abstract

Background/Objectives: Multimorbidity, defined as two or more chronic diseases co-occurring in an individual, has been linked to elevated healthcare utilization and out-of-pocket expenses. Yet, the barriers to healthcare access due to the health profile of multimorbid adults are underexplored. This study investigates the differences in delayed healthcare due to cost among adults with multimorbidity and those with one chronic disease condition. Methods: Data from the National Health Interview Survey from the years 2016–2018 and 2020–2021 were examined. The sample included 13,439 adults with at least one of the chronic disease conditions outlined by the US Department of Health and Human Services. Logistic regression models were used to estimate odd ratios of delayed healthcare due to cost among participants. Results: Multimorbid adults were 1.29 times more likely to delay healthcare compared to adults living with one chronic disease (*p* < 0.01). Other influencing factors include being female, Asian, unmarried, uninsured, age, worsening self-rated health, region, and poverty threshold of 100–199%. Conclusions: Our findings highlight the disparities in healthcare success experienced by adults living with multimorbidity and indicate the need for policymakers to implement targeted measures such as subsidized costs for comorbidities to alleviate the financial burdens experienced by this population.

## 1. Introduction

Healthcare delay is a significant factor influencing patients’ prognosis and disease management; therefore, examining healthcare delays among adults with chronic disease conditions is necessary to develop strategies that improve efficient disease management and positive health outcomes [1,2]. Adults living with multimorbidity, defined as the co-existence of two or more chronic diseases [3,4], are continuously burdened with higher financial costs due to their health profile, leading to delayed healthcare [5,6,7,8]. In 2018, about 51.6% of adults in the United States lived with at least one chronic disease, and over 27% were multimorbid [9]. Compared to people living with a single chronic health condition, multimorbid adults are more vulnerable and prone to health complications resulting in increased hospital admission, decreased physical activity, and even death if poorly managed, hence the need for continuous health monitoring [4,10].

Due to the increased need for consistent monitoring and increased level of healthcare utilization, multimorbid adults create financial burdens to both the healthcare system and themselves [5,6,7,8]. In the United States, 66% of the overall healthcare expenditure is attributed to the care of individuals with multimorbidity [11]. At the individual level, managing multiple chronic conditions is linked to elevated healthcare utilization and out-of-pocket expenses (OOPEs) [4,8], constituting 78% of all primary care hospital consultations in high-income countries [4]. Specifically, multimorbid adults encounter over twice the annual number of physician interactions compared to adults without multimorbidity, increasing with each additional chronic disease [12]. The relationship between healthcare utilization and the number of chronic disease conditions is nearly exponential, and this higher treatment burden places an increased financial strain on both the patient and their family [4,5,13]. Yet, the effect of this financial burden on the healthcare-seeking behavior of multimorbid adults is under-studied.

While numerous studies have explored the financial impact of multimorbidity on patients (for example, multimorbid adults were found to spend more on healthcare costs [14,15] and OOPEs compared to adults who had only one chronic disease [8,16]), there is limited research addressing the reduced healthcare access experienced by this population due to the financial burdens imposed by their health profile. Hence, the objective of this study is to fill this gap in the literature by investigating the differences in delayed healthcare needs due to cost among adults with multimorbidity in comparison to adults with a single chronic condition. The findings of this study highlight the impact of multimorbidity on delayed healthcare and provide practical, policy-focused strategies to enhance healthcare access for multimorbid adults, which aligns with the Healthy People 2023 goal to improve the health of the general population in the United States.

Having a chronic disease condition can potentially influence healthcare-seeking behavior due to financial constraints. Prior research has demonstrated that people living with chronic disease conditions such as chronic kidney disease (CKD), coronary heart disease (CAD), chronic obstructive pulmonary disease (COPD), asthma, and cancer have been found to delay healthcare due to financial limitations [17,18,19,20]. A few other studies disagree with this submission by documenting no association between chronic disease conditions and delayed medical care due to cost [1,21]; however, a study from 2012 found that among families with adults living with chronic disease conditions, even when individuals had health insurance, they were more inclined to postpone care when enrolled in high-deductible health plans (HDPDs) due to the increased financial burden associated with cost-sharing [22].

Although many adults in the United States are enrolled in a health insurance plan, the percentage of individuals enrolled in high-deductible health plans (HDHPs), which may lead them to delay healthcare, has increased over time [23,24]. HDHPs are plans with high deductibles, generally USD 1000 or more, and with lower premiums; however, enrolled participants pay more out-of-pocket before their insurance company begins to pay their share [24]. According to the Kaiser Family Foundation employer health benefits survey, in 2021, 28% of enrolled workers had an HDHP [25]. Theoretically, HDHPs were implemented to encourage more cost-conscious healthcare decisions among consumers; however, these plans lead patients to delay healthcare at the detriment of their health [23]. While the premiums on these plans appear to be cheaper, they obscure the higher OOPEs patients encounter when care is needed [26]. Low-income households were found to spend more than 20% of their disposable income on healthcare when enrolled in an HDHP [27].

Furthermore, research shows that chronic disease conditions are associated with poorer self-rated health ratings [28,29], and another study showed that older adults who rated their health poorly were more likely to delay healthcare [30]. However, while many studies have examined the financial impact of chronic disease conditions on delayed healthcare, the influence based on the number of chronic disease conditions remains unexplored. Using the National Health Interview Survey (NHIS) data, this study aims to fill this gap by examining the difference in delayed healthcare due to cost among adults with multimorbidity and adults with one chronic disease condition, thereby providing an understanding of the barriers encountered by multimorbid adults in healthcare utilization due to their health profiles.

## 2. Methods

### 2.1. Data Source

The National Health Interview Survey (NHIS) is a cross-sectional survey collected yearly with information principally based on non-institutionalized civilian population health in the United States. It is one of the primary data collection schemes by the National Center for Health Statistics (NCHS), used to observe disease trends and to monitor advancement to achieve national health objectives. The NHIS, initiated in 1957, has conducted surveys through the NCHS since 1960 to collect and examine data on a wide range of health-related topics to monitor the health of the inhabitants of the United States [31]. In the survey, geographically clustered sampling techniques, fashioned to obtain a nationally representative sample, are used to sample dwelling units each month and the data are collected continuously throughout the year via computer-assisted personal interviewing at the respondent’s home, while a telephone interview approach may be employed upon request by the respondent.

### 2.2. Study Populations

NHIS survey data between the years 2016 and 2021 were pooled and extracted from IPUMS to obtain a representative sample size, except for data for the year 2019, which were excluded due to incomplete data availability for the variables of interest. A total of 13,439 participants aged 18 years and older living with at least one chronic disease condition were included in this study. Out of 20 chronic diseases identified by the US Department of Health and Human Services, only 12 were available in the NHIS, of which 11 were included. Depression was excluded due to incomplete information for the years of study. Similar to other studies, this guideline was applied to produce a more standard approach to assessing the prevalence of chronic diseases [9] in the United States.

### 2.3. Ascertainment of Outcomes and Covariates

The dependent variable in this study is delayed care due to cost, which was measured based on a survey question asking respondents whether they “delayed medical care due to cost in the past 12 months”. Using this question, a binary variable (Yes and No) was constructed [32].

To measure the independent variable in this study, we included chronic disease conditions including arthritis, cancer, coronary heart disease, diabetes, chronic obstructive coronary disease (COPD), hepatitis, hypertension, stroke, weak or failing kidneys, current asthma, and high cholesterol. The respondents were asked if a doctor or another health professional ever told them they had any of the above-stated chronic conditions. The responses were no, yes, not in universe (n.i.u), unknown, and do not know. Based on their feedback, the respondents were classified as having either one, two, or more chronic disease conditions [9]. The covariates considered included race, sex, marital status, education, poverty threshold as a measure of wealth status, insurance status, age, region, and health status (Appendix A). The poverty threshold variable measures an individual’s income relative to the U.S. Census Bureau’s poverty threshold for the recorded year. It is a ratio that compares a person’s income to the poverty threshold and estimates the number of Americans living in poverty. People whose income falls below 100% of the threshold, are living in poverty, whereas individuals with an income above 100% are not living in poverty [33,34].

### 2.4. Statistical Analysis

Data analysis was conducted using Stata at different levels—univariate and multivariate. The univariate analysis involved presenting the frequency and percentages of the study variable as well as the mean and standard deviation of the age of the study respondents. At the multivariate level, binary logistic regression was employed to achieve the study objective. For the binary logistic regression, we fitted four models—Model 1 was an unadjusted model examining the differences in delayed healthcare due to cost between the multimorbid and single-chronic-condition groups. Model 2 additionally controlled for demographic factors, while in Model 3, we additionally controlled for socioeconomic factors, and, finally, Model 4 included all variables in addition to self-rated health. All analyses were weighted to provide a more accurate representation of the population.

## 3. Results

### 3.1. Sample Characteristics

Findings at the univariate level showed that 13.23% of the respondents delayed healthcare due to cost and 86.77% did not delay healthcare due to cost. Of all individuals living with chronic disease conditions, 68.03% had multimorbidity and 31.97% lived with a single chronic disease (see Table 1).

### 3.2. Binary Logistic Regression

Table 2 shows the binary logistic regression of the differences in delayed healthcare due to cost across single and multimorbid status, and, in Model 1, we found no significant difference in delayed healthcare due to cost outcomes between the two groups. In Model 2, adding demographic variables significantly influenced the outcome of interest. Compared to people living with one chronic disease, were male, married, white, or had an education at the professional or doctorate levels, people living with multimorbidity, were female, unmarried, from other races, or had education of less than a bachelor’s degree were more likely to delay healthcare due to cost. People who were Asian were less likely to delay healthcare than their white counterparts, and with increasing age, adults were less likely to delay healthcare.

Adults living with multimorbidity were 1.66 [95%CI: 1.40–1.96] times more likely to delay healthcare due to cost compared to people with a single chronic disease condition. Gender, marital status, race, age, region, and education also had significant effects on delaying healthcare due to cost. Female respondents were 1.29 [95%CI: 1.12–1.49] times more likely to delay healthcare due to cost compared to their male counterparts. Adults who were married were 1.37 [95%CI: 1.19–1.57] times more likely to delay healthcare due to cost compared to unmarried adults. People from other races were 1.35 [95%CI: 1.01–1.80] times more likely to delay healthcare, while Asian Americans were 51% less likely to delay healthcare due to cost compared to whites. With increasing age, adults were 2% less likely to delay healthcare due to cost. People residing in the Northcentral/Midwest, South, and West were 1.66 [95%CI: 1.33–2.07], 1.85 [95%CI: 1.51–1.28], 1.47 [95%CI: 1.18–1.83] times more likely to delay healthcare due to cost compared to those residing in the Northeast region, respectively. Respondents who had a bachelor’s degree were 1.88 [95%CI: 1.18–2.98] times more likely to delay healthcare compared to adults with a professional or doctoral degree.

Model 3 further included the socioeconomic factor variables. In this model, the differences in delayed healthcare among adults with multimorbidity and a single chronic disease remained significant. Insurance status and poverty threshold also significantly influenced healthcare delays. Compared to people with health insurance and people living below the 100% poverty level, people who were uninsured and living within the 100 to 199% poverty level were more likely to delay healthcare. Compared to adults living below 100% of the poverty threshold, adults living within 100 to 199% were 1.37 [95%CI: 1.13–1.66] times more likely to delay healthcare due to cost. Adults without health insurance were 5.75 [95%CI: 4.71–7.01] times more likely to delay healthcare due to cost compared to adults who had health insurance.

Model 4 additionally included the self-rated health variable. In this model, the differences in delayed healthcare among adults with multimorbidity and a single chronic disease remained significant. Each additional level decrease in self-rated health reflected an increase in the likelihood that a person would delay healthcare. With each unit’s decrease in self-rated health from excellent to poor, the participants were 1.58 [95%CI: 1.47–1.70] times more likely to delay healthcare.

## 4. Discussion

The significance of healthcare for the prognosis and management of chronic illness and the challenges associated with accessing such services from the perspective of cost warranted this study, which aimed to fill an important gap in the literature by examining the differences in delayed healthcare due to cost among adults living with multimorbidity and a single chronic disease. The findings show that being multimorbid significantly influences the likelihood of delayed healthcare due to cost compared to people with a single chronic disease condition controlling for sociodemographic and socioeconomic factors. The likelihood of people living with multimorbidity postponing needed healthcare could be linked to the fact that their care needs are quite complex and challenging for the healthcare team, patients, and their families, sometimes requiring multiple providers, medications, diagnostic evaluation, and various care plans, which entails high costs and could place a high financial burden on these individuals and their families. This finding is consistent with the existing body of research demonstrating the adverse effects of financial stress on this patient population [4,5,6,7,8], with the submission that adults living with multimorbidity were more inclined to postpone essential healthcare due to financial constraints compared to those who had a single chronic disease, thereby further supporting previous arguments related to the financial strain experienced by this population.

Furthermore, studies have revealed that individuals experiencing multimorbidity tend to encounter heightened occurrences of hospitalizations, greater medication use, extended hospital stays, and even require specialized care [35]. For multimorbid individuals, the possibility of their condition creating a financial hardship that compels them to defer necessary medical treatment due to cost constraints, potentially worsening their health conditions, is significant, which can consequently diminish their quality of life, life expectancy, and overall health outcomes. There has been an increase in the percentage of adults enrolled in HDHPs in the United States [23,24], which may be indicative of reasons why adults living with multimorbidity are more likely to delay healthcare due to cost. Although this population presents a greater need for healthcare, the increased financial burden they experience from high OOPEs seems to outweigh this need. This effect was also reflected in self-rated health results, which serve as a measure of health status [28].

### 4.1. Influencing Factors for Delayed Healthcare Due to Cost

Several other factors were also associated with delayed healthcare due to cost. As the self-rated health status depreciated from very good to poor, the rate of delayed healthcare due to cost increased considerably. People with poor self-rated health are more likely to live with more comorbidities, which invariably increases their healthcare expenses, thereby increasing the likelihood of delaying healthcare due to cost [36]. Our findings are similar to previous research that revealed that adults with poor or fair self-rated health status were significantly more likely to delay healthcare [37]. Our study also found that younger people were more likely to delay medical care due to cost than older people, which is consistent with the evidence in studies showing that middle-aged patients with chronic conditions are more likely to delay medical care and more likely to experience financial difficulties from medical care costs [17,37]. In the United States, healthcare is relatively expensive, with some left to out-of-pocket payments or increased health insurance premiums. While most young people are not eligible for government-sponsored insurance programs, older adults present with a higher number of chronic disease conditions and are eligible to participate in the government-sponsored Medicare insurance program [38], buffering the financial strain caused by multimorbidity.

Studies have shown that women are more likely to have multimorbidity [9,35] and therefore are expected to incur higher healthcare costs. However, in another study it was found that although more women were found to have multimorbidity, more men were seen in primary healthcare settings [39]. Similarly, another study found that although more women presented with multimorbidity, the overall incurred healthcare cost by gender was lower for women. This study found that women were more likely to delay healthcare due to cost compared to men [35]. According to the financial health network, men are less likely to be financially vulnerable compared to women [40]. This may explain why accessed care among women was lower. Furthermore, like other studies, married individuals were less likely to delay healthcare due to cost. Married people have stronger support systems in comparison to their unmarried counterparts and are more likely to experience less financial stress and may be on their spouse’s insurance plan [37]. In 2015, non-elderly married adults had the highest insurance coverage rates [41]; additionally, some other study found that married people were more likely to leave work and depend on their spouse’s insurance while receiving cancer treatments [42].

The Asian and Black populations were less likely to delay medical care due to cost compared to whites. The reason for this remains unclear. Minority groups, such as the Black population, may have a higher likelihood of residing below the poverty threshold, potentially leading to increased access to government-sponsored programs like Medicaid. In a 2016 study, African American population benefited immensely from the implementation of the Affordable Care Act (ACA) in 2014, granting them more access to healthcare than before [43]. Further investigation is necessary to delve into factors such as attitudes towards healthcare-seeking behavior within different racial groups to comprehend why Black and Asian populations are less likely to delay healthcare. Considering the region, people living in the West, South, and Northcentral/Midwest were considerably more likely to delay healthcare compared to those living in the Northeast. In a study among adults with CKD in the United States, it was found that individuals residing in the South experienced greater financial strain than those living in the Northeast region [17]. Some of the disparities observed may be influenced by income and socioeconomic status. Studies have indicated that compared to the Northeast, other regions such as the Midwest, South, and West experience a higher prevalence of poverty [44]. Consequently, individuals residing in these areas may experience higher levels of financial difficulties, potentially leading them to postpone or delay medical care because of cost.

Our findings also revealed that individuals with incomes ranging from 100 to 200% of the poverty line had a higher tendency to postpone medical treatment due to financial constraints. Although this population lives above the poverty line, they are ineligible for Medicaid insurance coverage, which could potentially expose them to greater financial burdens, increasing the likelihood of delaying healthcare services due to cost concerns [37,45]. Health insurance is designed to offset the higher healthcare cost, which is expected to significantly improve healthcare accessibility [46,47]. In this study, we found that not having health insurance coverage is associated with a higher likelihood of delaying healthcare, which is consistent with studies that have shown that in certain instances, individuals without health insurance may postpone seeking medical care until they secure employment that offers insurance benefits [48].

### 4.2. Limitations and Strengths

Our study is not without a few limitations. Firstly, the data from the National Health Interview Survey (NHIS) only cover non-institutionalized adults, excluding those in institutional settings like long-term care facilities. Consequently, the findings presented might not fully represent the impact of multimorbidity on healthcare access. Secondly, out of the 20 chronic disease conditions outlined by the US Department of Health and Human Services, only 11 were included in this analysis, and data on depression were omitted due to incomplete information; additionally, 2019 data were excluded, and the omission of depression and data from 2019 may lead to underestimation of effects. Thirdly, the data used in this study relied on self-reports, which inherently carry a risk of recall bias. Notwithstanding these constraints, a notable strength of this study lies in its substantial sample size and its utilization of a nationally representative cohort to examine the impact of multimorbidity on healthcare access among this population.

## 5. Conclusions

In this study, we examined healthcare delays due to cost disparities between adults with a single chronic disease and those with multiple chronic conditions. Our findings revealed that adults living with multimorbidity were more likely to delay healthcare because of financial constraints, reflecting the influence of financial limitations and their negative impact on health-seeking behavior, leading to delayed healthcare in this population. A lack of necessary care among multimorbid adults at the individual level can lead to further worse health outcomes and could increase the prevalence of multimorbidity at the population level while also increasing the incidence rate. For example, an adult with two poorly managed chronic disease conditions (e.g., diabetes and hypertension) could further develop a third disease (e.g., kidney disease), leading to increased health deterioration. Other contributing factors to delayed healthcare include marital status, gender, race, geographical region, income level, insurance status, and overall health status. These results underscore the necessity for policymakers to implement targeted measures such as subsidized costs for comorbidities aimed at alleviating the financial burdens experienced by this population, ultimately enhancing their access to necessary care, and leading to improved health outcomes. There are several healthcare programs and plans in the United States such as health management organizations [HMOs] and Preferred Provider Organizations [PPOs], among others; such plans should consider the fact that the healthcare experiences of multimorbid individuals might be different from those of individuals living with single chronic; hence, proper accommodation should be made. In addition, it will be highly beneficial for individuals living with multimorbidity to have Medicaid and Medicare programs that consider their plights, especially those who do not currently qualify for any of these programs. Future studies may consider assessing the influence of multimorbidity on healthcare access within disease clusters more likely to occur simultaneously, variations in delayed healthcare by insurance provider types, and if delayed healthcare in this population will translate into forgone healthcare due to cost.

## Figures and Tables

**Table 1:** Descriptive statistics of study population N = 13,439.

Characteristics	Mean ± SD or N (%)
Delayed healthcare due to cost	
No	11,660 (86.76)
Yes	1779 (13.24)
Chronic disease status	
Single chronic disease	4294 (31.95)
Multimorbid	9145 (68.05)
Sex	
Male	4851 (36.10)
female	8588 (63.9)
Marital Status	
Married	5561 (41.38)
Not married	7878 (58.62)
Race	
White	10,643 (79.19)
Black	1748 (13.01)
American Indian and Alaskan Native (AIAN)	167 (1.24)
Asian	418 (3.11)
Others	463 (3.45)
Age	52.11 ± 17.72
Region	
Northeast	2309 (17.18)
Northcentral/Midwest	3126 (23.26)
South	4611 (34.31)
West	3393 (25.25)
Education	
Less than bachelor	8997 (66.95)
Bachelors	2648 (19.70)
Masters	1335 (9.93)
Professional/doctoral	459 (3.42)
Poverty threshold	
Less than 100%	2234 (16.62)
100–199%	2592 (19.29)
200% and above	8613 (64.09)
Insurance status	
Insured	12,646 (94.10)
Not insured	793 (5.90)
Self-rated health	2.77 ± 1.12

**Table 2:** Odds ratios for delayed healthcare due to cost among adults with multimorbidity.

Variables	Model 1	Model 2	Model 3	Model 4
Chronic disease status (Ref = One chronic condition)				
Multimorbid	1.12 [0.98–1.29]	1.66 *** [1.40–1.96]	1.72 *** [1.45–2.04]	1.29 ** [1.08–1.54]
Sex (Ref = Male)				
female		1.29 *** [1.12–1.49]	1.36 *** [1.17–1.58]	1.34 *** [1.15–1.56]
Marital status (Ref = Married)				
Not married		1.37 *** [1.19–1.57]	1.21 ** [1.04–1.40]	1.18 * [1.02–1.37]
Race (Ref = White)				
Black		0.83 [0.68–1.02]	0.79 * [0.63–0.98]	0.77 * [0.62–0.96]
AIAN		1.06 [0.62–1.83]	0.71 [0.37–1.33]	0.64 [0.34–1.21]
Asian		0.49 *** [0.32–0.75]	0.52 ** [0.34–0.80]	0.52 ** [0.34–0.80]
Others		1.35 * [1.01–1.80]	1.32 [0.99–1.82]	1.28 [0.94–1.75]
Age		0.98 *** [0.98–0.98]	0.98 *** [0.98–0.99]	0.98 *** [0.98–0.98]
Region (Ref = Northeast)				
Northcentral/Midwest		1.66 *** [1.33–2.07]	1.56 *** [1.24–1.95]	1.50 *** [1.20–1.89]
South		1.85 *** [1.51–2.28]	1.55 *** [1.25–1.92]	1.49 *** [1.20–1.84]
West		1.47 *** [1.18–1.83]	1.42 *** [1.14–1.78]	1.40 ** [1.12–1.75]
Education (Ref = Professional/Doctoral)				
Less than bachelor		1.88 ** [1.19–2.98]	1.50 [0.94–2.39]	1.12 [0.70–1.81]
Bachelors		1.71 * [1.06–2.78]	1.70 * [1.04–2.76]	1.51 [0.92–2.49]
Masters		1.45 [0.88–2.34]	1.48 [0.89–2.45]	1.39 [0.83–2.32]
Poverty threshold (Ref = Less than 100%)				
100–199%			1.37 ** [1.13–1.66]	1.47 *** [1.21–1.80]
200% and above			0.88 [0.72–1.07]	1.10 [0.90–1.35]
Insurance status (Ref = Insured)				
Not insured			5.75 *** [4.71–701]	5.96 *** [4.84–7.34]
Health status (Ref = Excellent)				1.58 *** [1.47–1.70]
n	13,439	13,439	13,439	13,439

Note: 95% Confidence Intervals in parentheses, * *p* < 0.05, ** *p* < 0.01, *** *p* < 0.001.

## Data Availability

This study utilized publicly available data from the National Health Interview Survey at IPUMS NHIS: vars by group.

## Appendix A

**Table A1:** Covariates’ coding scheme.

Variables	Code
Sex	Male = 0 Female = 1
Marital status	Not married = 1 Married = 0
Education	Less than bachelor = 1 Bachelors = 2 Master = 3 Professional/doctoral = 4
Poverty threshold	Less than 100% = 1 100–199% = 2 200% and above = 3
Insurance status	Not insured = 1 Insured = 0
Age	18 and above
Region	Northeast Northcentral/Midwest South West
Health status	Excellent = 1 Very good = 2 Good = 3 Poor = 4 Very poor = 5
